# *Bacillus velezensis* YC7010 Enhances Plant Defenses Against Brown Planthopper Through Transcriptomic and Metabolic Changes in Rice

**DOI:** 10.3389/fpls.2018.01904

**Published:** 2018-12-21

**Authors:** Md. Harun-Or-Rashid, Hyun-Jin Kim, Seon-In Yeom, Hyeon-Ah Yu, Md. Maniruzzaman Manir, Surk-Sik Moon, Yang Jae Kang, Young Ryun Chung

**Affiliations:** ^1^Division of Applied Life Science (BK21 Plus), Plant Molecular Biologyand Biotechnology Research Center, Gyeongsang National University, Jinju, South Korea; ^2^Division of Entomology, Bangladesh Agricultural Research Institute, Rangpur, Bangladesh; ^3^Division of Applied Life Science (BK21 Plus), Department of Food Science and Technology, College of Agriculture and Life Sciences, Gyeongsang National University, Jinju, South Korea; ^4^Department of Agricultural Plant Science, Institute of Agriculture and Life Science, Gyeongsang National University, Jinju, South Korea; ^5^Napro Biotech, Gongju, South Korea; ^6^Department of Chemistry, Kongju National University, Gongju, South Korea

**Keywords:** *Bacillus velezensis* YC7010, brown planthopper, induce systemic resistance, rice, secondary metabolites

## Abstract

Brown planthopper (BPH; *Nilaparvata lugens* Stål) is one of the most serious insect pests, which reduce rice yield remarkably in many rice-growing areas. A few plant growth-promoting rhizobacteria induce systemic resistance against herbivorous insects. Here we show that root drenching of rice seedlings with an endophytic strain *Bacillus velezensis* YC7010 enhanced defenses against BPH. Based on high-throughput transcriptome analysis, systemic resistance against BPH was induced by *B. velezensis* YC7010 via salicylic acid (SA)- and jasmonic acid (JA)-dependent pathways. Increased leaf contents of secondary metabolites, tricin and *C*-glycosyl flavone and cell-wall contents of lignin and cellulose were the key defense mechanisms inducing resistance against BPH during the three-way interaction. This study shows for the first time that chemical changes and strengthening of physical barriers play important roles simultaneously in plant defense against BPH in rice by the endophytic bacteria. This defense was induced by lipopeptides including a novel bacillopeptin X.

## Introduction

The brown planthopper (BPH; *Nilaparvata lugens* Stål) is one of the major damaging insect pests of rice (*Oryza sativa* L.) with a typical herbivorous phloem-sucking habit. Continued BPH infestations normally reduce photosynthates and thereby hinder plant growth, which causes the wilting of tillers, known as “hopper-burn” and heavy yield losses in susceptible rice varieties (Liu et al., 2008). Their feeding also spreads certain rice viruses such as grassy stunt virus and ragged stunt virus, which are serious problems associated with rice production in cultivated areas of Asia (Suzuki et al., 2015). The main control methods for BPH involve an integrated pest management strategy via application of chemical insecticides and/or cultivation of resistant varieties. However, breakdown of plant resistance to BPH and BPH resistance to chemical insecticides often results in increased levels of infestation (Hu et al., 2016). Abuse or misuse of chemicals results in the destruction of the natural balance of BPH predators that control the BPH population and also cause insect resurgence (Tanaka et al., 2000). During the last decades, development of integrated pest management approaches based on natural resources and traits such as host resistance and biological control was used to reduce the role of chemical pesticides in insect control strategy (Hong-xing et al., 2017). Biological control using microorganisms or antibiotics offers a good alternative to chemical control and provides efficient control with little or no natural environmental hazard (Li et al., 2014). Use of plant growth-promoting rhizobacteria (PGPR) is a potential strategy for successful biological control (Nelson, 2004).

Plant growth-promoting rhizobacteria living in association with the roots of higher plants increase host adaptation under biotic and abiotic stresses to increase their growth and improve stress tolerance (Niazi et al., 2014). PGPR may activate molecular and physiological alterations in plants mediating enhanced plant defense to pathogens as well as insect pests via induced systemic resistance (ISR) (Van Oosten et al., 2008; De Vleesschauwer and Höfte, 2009; Pineda et al., 2010; Rashid et al., 2017). The endophytic bacteria colonize the rhizosphere effectively by competing with the native soil microbiota (Garbeva et al., 2004). Recent studies investigating the enormous diversity and abundance of endophytic bacteria in plants raised interest in the development of endophytic bacteria as biological control agents (D’Alessandro et al., 2014). Plant defense systems also were triggered by bacterial endophytes through a variety of pathways resulting in the alteration of host plant physiology accordingly (Compant et al., 2010; Wielkopolan and Obrêpalska-Stêplowska, 2016). The mechanisms of plant defense against plant pathogens induced by rhizobacteria are well investigated, but the mechanisms against herbivorous insects are not yet elucidated (van Loon et al., 1998; De Vleesschauwer and Höfte, 2009; Pineda et al., 2010).

To initiate plant defense mechanisms against herbivorous insects and simultaneously establish associations with PGPR, plants use phytohormones such as ethylene (ET), jasmonic acid (JA), and salicylic acid (SA) as signal molecules to coordinate their immune responses (Pieterse et al., 2012; Zamioudis and Pieterse, 2012). Differences in defense mechanisms were observed depending on the feeding behavior of chewing or sucking insects (Pineda et al., 2010). Sucking insects such as phloem-feeding whiteflies and aphids that cause little injury to plant foliage are perceived as pathogens and primarily activate SA-dependent and to a certain extent JA/ET-dependent signaling pathways to ISR (Walling, 2000; Li et al., 2006; Zarate et al., 2007). JA-mediated defenses are known to be activated against chewing insects (Kessler et al., 2004; De Vos et al., 2005; Zheng et al., 2007; Howe and Jander, 2008; Van Oosten et al., 2008). ISR against whiteflies by *Bacillus subtilis* strain BEB-DN (BsDN) appear to be a combination of JA-dependent and -independent responses (Valenzuela-Soto et al., 2010).

A wide range of elicitors such as volatile compounds and lipopeptides produced by PGPR are perceived by host plants to activate the ISR in defense responses and the related metabolic changes in plants (Ryu et al., 2004; Ongena et al., 2007; Henry et al., 2011). After early perception of pest infestation in host plants, various defense-related metabolites such as phytoalexin and phenolic compounds are accumulated in association with ISR mechanisms (De Vleesschauwer et al., 2008). The biosynthesis of a wide variety of phenolic compounds including flavonoids occurs via phenylpropanoid pathway in the plant defense against herbivores (McMullen et al., 2009; Rashid and Chung, 2017). In the biosynthesis of phenolics, phenylalanine ammonia-lyase (PAL) is the initial enzyme in the phenylpropanoid pathway inducing plant resistance (Pellegrini et al., 1994; Polle et al., 1994). Oxidative enzymes such as peroxidase (POD) and polyphenol oxidase (PPO) are also important in the formation of other oxidative phenolic compounds and lignin that are associated with defense barriers for strengthening the cell structure against plant pathogens and insects (Avdiushko et al., 1993; McMullen et al., 2009; Cesarino et al., 2012). The increased content of both lignin and cellulose induced by foliar endophytic fungus plays an important role in reduction of damage induced by pathogens and herbivores in a tropical tree, *Theobroma cacao* (Mejía et al., 2014). In addition to defense-related chemical compounds, plant proteins and lectins are known to exhibit insecticidal activity. Orysata, a jacalin-related lectin from rice protects host plant against both biting-chewing beet armyworm and piercing-sucking aphids (Vandenborre et al., 2011; Al Atalah et al., 2014).

Although BPH is one of the major insect pests in rice, little is known about the molecular mechanism of interaction with the host and its attack. Yuan et al. (2005) reported the upregulation of macromolecule degradation and plant defense genes, whereas the genes for photosynthesis and cell growth were downregulated by BPH infestation of the susceptible rice cultivar. OsLOX1, JA biosynthesis gene, induced tolerance of the rice plant to wounding and BPH attack, and the SA and ET signaling pathways were positively correlated with resistance to BPH attack in rice (Wang R. et al., 2008; Zhou et al., 2009; Lu et al., 2011). However, genes related to wounding, oxidative and pathogen stresses overlapped extensively between BPH-resistant and susceptible rice lines (Wei et al., 2009). RNA sequencing analysis of rice with BPH infestation revealed that genes encoding JA/ET signaling, receptor kinase, MAPK cascades, Ca^2+^ signaling and protein posttranslational modifications play an important role in the defense mechanism (Lv et al., 2014). In addition, secondary metabolites including flavonoids, phenylpropanoids, polyphenols, oxalic and silicic acid and phenolics also play essential roles in rice resistance to BPH (Yoshihara et al., 1979, 1980; Stevenson et al., 1996; Zhao et al., 2004; Schwachtje and Baldwin, 2008; Liu et al., 2010). Among these compounds, tricin is one of the flavonoids especially shown to induce resistance against BPH in a resistant rice cultivar (Bing et al., 2007).

In this study, the molecular mechanism and biochemical changes induced by the endophytic strain of *B. velezensis* YC7010 were elucidated using an integrated analysis of the transcriptome and the metabolome of rice-endophytic bacterial interaction to defense against BPH in rice. Endophytic stain *B. velezensis* YC7010 was isolated from rice root with plant growth promoting, antibiotic and induced systemic resistance to soil-borne rice bacterial, fungal pathogens, and insect (Chung et al., 2015; Hossain et al., 2016; Rashid et al., 2017). The chemical structure of a novel compound inducing ISR against BPH was also determined. The results provide novel insights into the dynamic molecular changes of the three-way interaction between rice, bacteria and BPH in terms of defense response against the destructive insect pest.

## Materials and Methods

### Preparation of Rice Seedlings and Rearing of BPH

Rice (*Oryza sativa* L. ssp. *japonica* cv. Dongjin) seeds were surface-sterilized with 70% ethanol for 5 min followed by 1.2% sodium hypochlorite (NaOCl) solution for 5 min and rinsed with sterile distilled water several times. The seeds were left in water at 30°C for 3 days in dark with daily change of water. The germinated seeds were sown in commercial nursery potting soils and incubated in a growth chamber at 30°C (light; 200 μmol m^-2^ sec^-1^ and 16 h-light/8 h-dark regime) (Hossain et al., 2016). The 2-week-old seedlings were transplanted into the plastic pots (11.5 cm × 10 cm × 7.5 cm^3^) containing about 150 g of sterilized nursery soils and cultivated until completion of tests. The BPH population was collected from the rice paddy fields located at Jinju, Korea. The BPH insects were reared on susceptible rice seedlings Taichung Native-1 (TN1) in the growth chamber at 30°C with 80% relative humidity.

### Induction of Systemic Resistance to BPH by *B. velezensis* YC7010

The endophytic *B. velezensis* YC7010 isolated from rice root was used to induce systemic resistance against BPH. The bacterial strain was cultivated in one-tenth strength tryptic soy broth (1/10 TSB, Bacto™, Sparks, MD, United States) on a rotary shaker (160 rpm) at 28°C for 48 h. Cells were centrifuged at 6,000 *g* for 15 min and suspended in a buffer solution (10 mM MgSO_4_) to adjust to 2 × 10^7^CFU ml^-1^ for use (Chung et al., 2015). Three-week-old rice seedlings were drenched with 15 mL bacterial suspension on the rhizosphere in each pot. An equal volume of 10 mM MgSO_4_ was drenched as control. At 5 days after bacterial inoculation, both treated and control plants were infested with 15 s instar nymphae per plant seedling and covered using fine and light-transmitting mesh. Seven days post-infestation, the BPH severity score of each seedling was assessed using a score of 0, 1, 3, 5, 7 or 9 (Huang et al., 2001). Each treatment had fifteen replicates. Experiments were conducted three times.

### Plant Materials and RNA Extraction

Three-week-old rice seedlings were used for RNA extraction after treatment with the bacterial strain and BPH (Supplementary Figure S1). A total of 10 s instar nymphae were laid on both bacteria-treated and untreated control plants at 5 days after bacterial treatment. The leaves of 15 treated and untreated rice plants were collected as a mixed sample at 48 h post infestation (hpi) with or without BPH and BPH was removed from the leaves before samples collection. Four samples were collected from bacterial untreated without (Control) or with (Control + BPH) BPH and bacterial treated without (YC7010) or with (YC7010 + BPH) BPH infested rice plants. All experiments were conducted in three biological replicates. Collected leaves were frozen and ground in liquid nitrogen to a fine powder and the total RNA was extracted from the samples using RNA extraction kit (Qiagen RNeasy Plant Mini Kit) according to the manufacturer’s instructions and the RNA-seq paired end libraries were constructed using three biological replicates at 48 hpt using Illumina TruSeq RNA Sample Preparation Kit v2 (catalog #RS-122-2001, Illumina, San Diego, CA, United States) according to the manufacturer’s instructions.

### Transcriptome Analysis

Starting with total RNA, mRNA purified using poly (A) selection or rRNA depleted, then RNA chemically fragmented and converted into single-stranded cDNA using random hexamer priming. Next, the second strand is generated to create double-stranded cDNA. Library construction begins with generation of blunt-end cDNA fragments from ds-cDNA. Then A-base added to the blunt-end in order to make them ready for ligation of sequencing adapters. After the size selection of ligates, the ligated cDNA fragments which contain adapter sequences are enhanced via PCR using adapter specific primers. The library was quantified with KAPA library quantification kit (Kapa Biosystems KK4854) following the manufacturer’s instructions. All constructed libraries with an insert length of 101 bp were sequenced using Illumina HiSeq 2000 (Illumina Inc., San Diego, CA, United States; Supplementary Table S1). Sequence data whose quality of bp was higher than Q ≥ 20 were extracted by SolexaQA (Cox et al., 2010). Trimmed reads were mapped using the RNA-seq mapping algorithm implemented in bowtie2 (v2.1.0) software (Langmead et al., 2009) to the transcripts of *O. sativa* MSU release 7 from Phytozome V.10^1^ (Ouyang et al., 2006) allowing all alignment with a maximum of two mismatches. The number of mapped clean reads for each gene was counted and normalized with DESeq package in R (Anders and Huber, 2010) to avoid bias due to different sequencing amounts. The RNA-Seq sequences of this test have been deposited at the NCBI and the accession number is SAMN10364481∼86.

### Analysis of Differentially Expressed Genes

Differentially expressed genes (DEGs) were identified by a ≥2-fold change in the number of mapped reads, a binomial test with a false discovery rate (FDR) ≤ 0.01, and a read count ≥ 1,000 between samples. The FDR was applied to identify the threshold *p*-value for multiple tests and was calculated using DESeq (Anders and Huber, 2010). All correlation analysis and hierarchical clustering were performed using AMAP library in R (Lucas, 2014). For pathway analysis, we mapped all DEGs using the MapMan package (Thimm et al., 2004) with the Osa_MSU_v7 mapping file and latest pathways downloaded from the official web site^2^. For each DEG gene list, down-regulated and up-regulated genes were annotated using Gene Ontology (GO) based on the similarity of protein sequences in the GO database (Ashburner et al., 2000). The number of genes assigned in each GO term was counted using in-house scripts of SEEDERS Co.

### Real-Time qPCR

Sixteen genes of MapMan pathway classifications were selected for validation using real-time qPCR. Primer sets were designed with the OligoPerfect™ Designer software and the primers used in real-time qPCR are provided in Supplementary Table S2 as supporting information. Quantitative real-time PCR was performed using the CFX96^®^Real-Time PCR Detection System (Bio-Rad^3^). OsAct1 was amplified and used as an internal positive control. All results had three biological replicates.

### Assay of Defense-Related Enzyme Activity

Three defense-related enzymes including PAL, PPO, and POD were measured using 3-week-old rice seedlings treated with the bacterial strain and BPH. Both treated and untreated control plants were exposed to a total of 10 BPH at 5 days after bacterial treatment. For enzyme assay, the leaves of 15 treated and untreated rice plants were collected as a mixed samples at 0, 24, and 48 hpi of BPH. To measure the activities of PAL (Qin and Tian, 2005), 3 g of rice samples from each treatment were homogenized with polyvinyl pyrrolidone (PVP) ( 0.5 g) and sodium borate buffer (30 ml, 50 mmol L^-1^, pH 8.8, containing 5 mmol L^-1^ β-mercaptoethanol) and crushed by tissue grinder at 4°C. Grinding samples were centrifuged at 15,000 ×*g* for 30 min at 4°C, and the supernatant was collected for PAL assays. Enzyme extract (1 ml) was incubated with borate buffer (2 ml, 50 mmol L^-1^, pH 8.8) and L-phenylalanine (0.5 ml, 20 mmol L^-1^) at 37°C for 60 min. The reaction was stopped with HCl (0.1 ml, 6 mol L^-1^). The PAL activity was determined by the absorbance change at 290 nm. To measure the activities of PPO and POD (Chen et al., 2000), 3 g of rice samples from each treatment were homogenized with sodium phosphate buffer (30 ml, 0.1 mol L^-1^, pH 6.4) and PVP (0.5 g) and ground at 4°C. The mixture were centrifuged at 15,000 ×*g* for 30 min at 4°C, and the supernatant were used for PPO and POD assays. The PPO activity were determined by adding 1 ml of enzyme preparation to 2 ml of catechol as a substrate, and the change were measured immediately in absorbance at 398 nm (A398). The POD activity was measured by guaiacol as a substrate. The reaction mixture contained of crude extract (2 ml), buffer (1 ml), and guaiacol (1 ml). The reaction mixture was incubated for 30 min at 30°C before added of H_2_O_2_ (1 ml). Absorbance was calculated at 460 nm (A460). All results had three biological replicates.

### Measurement of Chlorophyll, Lignin and Cellulose Content

To measure chlorophyll, lignin and cellulose contents in leaves or stem, rice seedlings were collected at 5 d after BPH infestation from 15 bacteria-treated and untreated plants and the contents were determined. The total chlorophyll content was determined as described previously (Rashid et al., 2017). Lignin was measured quantitatively according to Lange et al. (1995). Extraction of cell wall materials and determination of cellulose contents were conducted as described previously (Updegraff, 1969; Li et al., 2003). All results had three biological replicates.

### Metabolomics Analysis and Data Processing

A metabolomics analysis of rice interaction with BPH and bacteria was conducted by collecting the leaves of 3-week-old rice seedlings with or without BPH at 48 h after bacterial infection. The samples were collected from 15 rice plants for each treatment. The collected samples were homogenized with 80% aqueous methanol with terfenadine as an internal standard using a bullet blender (Next Advance, NY, United States) to extract metabolites. After centrifugation, the extracted metabolites in supernatants were analyzed using an ultra-performance liquid chromatography-quadrupole/time-of-flight mass spectrometry (UPLC-Q-TOF MS) system (Xevo™ G2-S; Waters, Milford, MA, United States). The samples were injected into an Acquity UPLC BEH C18 column (2.1 mm × 100 mm, 1.7 μm; Waters) at a column temperature of 40°C. The mobile phase consisted of water with 0.1% formic acid in water (A) and ACN with 0.1% formic acid (B) at a flow rate of 0.35 mL/min for 15 min. The eluents were analyzed using a Q-TOF MS with positive electrospray ionization (ESI). The scan range of TOF MS data was 100 to 1500 m/z with a scan time of 0.2 s. The capillary and sampling cone voltages were set at 3 kV and 40 V, respectively. The desolvation flow rate was 900 L/h at a temperature of 300°C and source temperature set to 100°C. Leucine-enkephalin ([M+H] = 556.2771) was used as a reference compound for lack mass at a frequency of 10 s. Quality control (QC) samples prepared by mixing equal amounts of samples were injected periodically. The MS/MS spectra were collected in the m/z 50–1500 using a collision energy ramp from 10 to 45 eV. All MS data, including retention time, *m/z*, and ion intensity, obtained by UPLC-Q-TOF MS, were extracted with MarkerLynx software (Waters) for data processing. The peaks were collected using peak-to-peak baseline noise of 1, noise elimination of 6, a peak-width at 5% height of 1 s and an intensity threshold of 10,000. The metabolite data were evenly arranged with a mass window (0.05 Da) and a retention time window (0.2 min). All data was normalized to the internal standard. Identification of the metabolites was based on Unifi software (Waters) with various online databases.

### Structure Determination of Bacterial Compounds With ISR Activity to BPH

#### General Experimental Procedures

The NMR spectra were recorded on a Varian Mercury 400 spectrometer (Varian Inc) with standard pulse sequences operating at 400 MHz for ^1^H-NMR and 100 MHz for ^13^C-NMR. Chemical shifts, measured in ppm, were referenced to solvent peaks (δ_H_ 2.50 and δ_C_ 39.5 for DMSO-*d_6_*). HR-TOF-MS spectra were recorded in the positive ESI mode on a Waters Synapt G2 at the Korea Basic Science Institute. Flash column chromatography was performed on a C18 silica gel column (Cosmosil 75C18-Prep, Nacalai Tesque, Koyoto, Japan; 100 id × 180 mm) using a 10% stepwise gradient elution of increasing MeOH concentration in H_2_O (2.0 L each). Medium pressure liquid chromatography (MPLC) was carried out using silica gel column (230–400 mesh, 25 id × 110 mm) with a Yamazen pump 540 eluting with a EtOAC-MeOH gradient (1:0, 1:1, and 0:1, 500 mL each). Preparative C18 HPLC was conducted on a Gilson 321 pump equipped with a photodiode array UV detector UV/VIS-151 using Varian Dynamax HPLC column (21.4 id × 250 mm, Microsorb 100–5 μm C18, 90–100% aqueous MeOH) utilized UV detection of peaks at 220 nm, flow rate of eluent at 7 mL/min, and gradient elution for 80 min.

Extraction and isolation of bacterial compounds are detailed in the Supplementary Table S3. To determine the ISR activity of the active compound to BPH, surface-sterilized seeds with 70% ethanol for 5 min followed by 1.2% sodium hypochlorite (NaOCl) solution for 5 min and rinsed with sterile distilled water several times were sown on microwell plates (2 cm × 2 cm) containing half-strength MS solid media with 0.8% (w/v) agar. The seeds were cultivated for 2 weeks in a growth chamber under the same conditions as described in the preparation of rice seedlings. The roots of seedlings were treated with 100 μl solution of purified compound (bacillopeptin A, bacillopeptin B and bacillopeptin X; 100 μg ml^-1^) dissolved in distilled water and an equal volume of water was used to drench the roots as control. Five days after chemical treatment, the treated seedlings were transplanted into pots (11.5 cm × 10 cm × 7.5 cm) containing about 150 g of sterilized nursery soils and infested with 15 second instar nymphae per seedling at 3 days after transplant. Seven days after BPH infestation, the severity score was assessed as described previously. Each treatment had fives replicates. Experiments were conducted three times.

### Statistical Analysis

All data were subjected to the analysis of variance (ANOVA) technique and the mean differences were estimated by Tukey’s HSD and Student’s *t*-test using statistical software, SPSS 17 (SPSS Inc. Chicago) and Sigma plot (version 12).

## Results

### Induction of Systemic Resistance to BPH by *B. velezensis* YC7010

**FIGURE 1:**
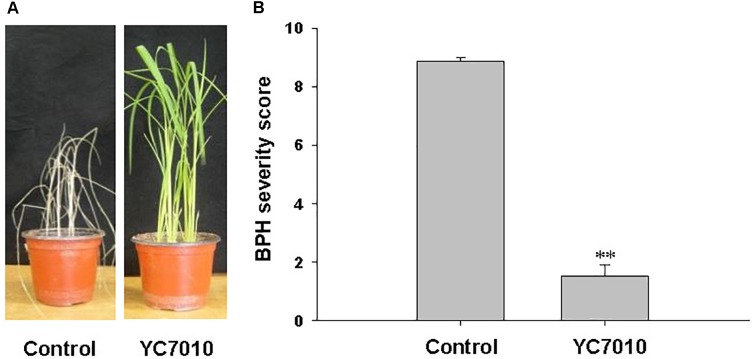
Induction of systemic resistance to BPH in rice by inoculation of *Bacillus velezensis* YC7010. Bacterial suspension (2 × 10^7^ CFU/mL) was drenched to the rhizosphere of 3-week-old rice seedlings while 10 mM MgSO_4_ solution was used as negative control. Five days later, plants were infested with 15 s instar nymphae per plant seedling and covered using a fine, light-transmitting mesh. **(A)** Rice seedlings imaged after BPH infestation. **(B)** BPH severity score was determined at 7 days after infestation. Data were analyzed by independent Student’s *t*-tests. Bars represent mean values ± standard error (SE). Statistical significance for treatment effects is marked ^∗∗^*P* < 0.01. Each treatment had fifteen replicates. All experiments were conducted in triplicate with similar results.

The endophytic strain *B. velezensis* YC7010 was tested for its capacity to trigger ISR to BPH in rice. Seven days after infestation with 15 s instar nymphaea, the rice seedlings root-drenched with the bacterial suspension were healthy, whereas untreated control plants showed symptoms of severe stem chlorosis, leaf wilting and even death of the whole plant (Figure 1A). Bacteria-treated plants exhibited a significantly (*P* < 0.01) lower BPH severity score 1.53 compared with that of untreated control 8.87 (Figure 1B). The results showed that rice seedlings treated with *B. velezensis* YC7010 induced resistance against infestation by BPH.

### Transcriptome Analysis of Rice Treated With *B. velezensis* YC7010 in Response to BPH Infestation

**FIGURE 2:**
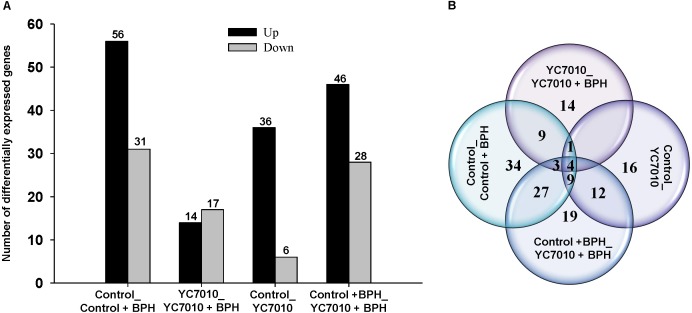
Differentially expressed genes (DEGs) induced in rice by *B. velezensis* YC7010 and BPH infestation. **(A)** Comparison of upregulated and downregulated differentially expressed genes exposed to four treatments with (YC7010) or without (Control) inoculation of bacteria and with (BPH) or without (Control) infestation. **(B)** Venn diagram displaying all the DEGs.

Gene expression profiles of rice seedlings treated and untreated with bacteria were investigated using deep RNA sequencing to identify defense-related genes in rice in response to BPH infestation to elucidate the molecular mechanism of systemic resistance induced by *B. velezensis* YC7010. RNAs extracted from rice samples at 48 hpi of BPH in inoculated and untreated control plants were sequenced and DEGs of samples were analyzed. In the study, we defined DEGs as the transcripts showing at least a twofold change of the FPKM (fragments per kilo base of exon per million fragments mapped) (log2FC ≥ 1 or log2FC ≤-1) and a *P*-value < 0.01. In total, 134 DEGs were detected by comparing the four treatments with (YC7010) or without (Control) inoculation of bacteria and with (BPH) or without (Control) infestation of insects (Supplementary Table S4). BPH infestation altered the gene expression of the untreated plant significantly, including upregulation of 56 genes and downregulation of 31 genes (Control_Control + BPH). After root drenching of rice seedlings with the bacteria, there were 42 differentially expressed genes, including 36 upregulated genes and 6 downregulated genes compared with the untreated control. Following bacterial treatment of rice roots, BPH infestation showed fewer DEGs than in other comparisons (YC7010_YC7010 + BPH). There were 31 DEGs, including 14 upregulated genes and 17 downregulated genes. Comparison of DEGs between bacteria-treated and untreated plants infested with BPH (Control + BPH_YC7010 + BPH) revealed significant changes in gene expression of the infected plants, including 46 upregulated genes and 28 downregulated genes (Figure 2A). Moreover, the number of DEGs between Control_Control + BPH and YC7010_YC7010 + BPH, YC7010_YC7010 + BPH and Control_YC7010, Control_YC7010 and Control + BPH_YC7010 + BPH and Control_Control + BPH and Control + BPH_YC7010 + BPH were 17 (9+1+4+3), 5 (1+4), 25 (12+9+4), and 43 (4+9+27+3), respectively (Figure 2B).

**FIGURE 3:**
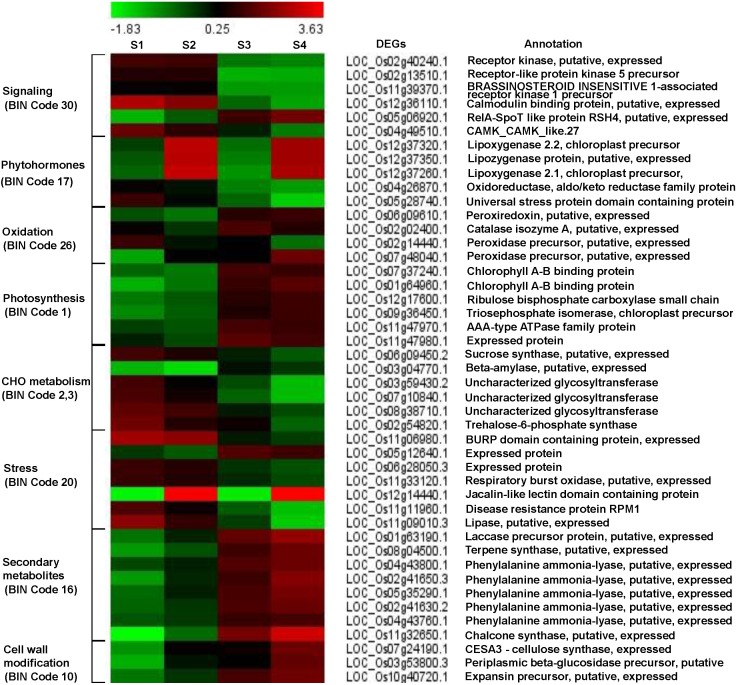
Fold change patterns of representative pathway genes. The color key represents FPKM normalized log2 transformed counts. Red indicates upregulated DEGs and blue denotes downregulated DEGs. Each column shows a comparison and each row represents a gene. S1, S2, S3, and S4 represent comparisons of Control_Control + BPH, YC7010_YC7010 + BPH, Control_YC7010, and Control + BPH_YC7010 + BPH, respectively.

Differentially expressed genes were imported into the MapMan software and the classification based on the hierarchical functional categories, BINs and sub-BINs is listed in Supplementary Table S5 and provided in Supplementary Figure S2. The overview analysis using the MapMan software grouped 125 DEGs into 25 BINs. This global overview suggested that about 74.4% of 93 genes grouped into MapMan BINs were classified under 9 major functional classes with 5 or more genes. The BIN 1 representing ‘Photosynthesis’ group comprised the most number of DEGs (24) (Supplementary Figure S2). Based on the MapMan software and GO function pathway analyses, we identified several genes related to major pathways such as early defense signaling, oxidative stress, photosynthesis, carbohydrate, secondary metabolites and cell-wall modification were investigated in detail to understand the defense mechanisms and the functional significance underlying the three-way interaction (Figure 3 and Supplementary Table S6).

### Early Defense Signaling Induced by *B. velezensis* YC7010 and BPH Infestation in Rice

Six genes were involved in perception and signal transduction pathways in which Ca^2+^ played an important role as a second messenger. These included LRR family protein receptors such as protein kinase EXS precursor (LOC_Os02g40240.1), receptor-like protein kinase 5 (LOC_Os02g13510.1) and brassinosteroid-insensitive 1-associated receptor kinase 1 precursor BAK1 (LOC_Os11g39370.1). The three genes were upregulated in both bacteria-inoculated and control plants after BPH infestation, but were downregulated in BPH-infested plants treated with bacteria compared with the untreated BPH infested plant (Figure 3). Ca^2+^ signaling-related genes such as calmodulin binding protein (LOC_Os12g36110.1) and calmodulin-dependent protein kinases (LOC_Os04g49510.1) were upregulated in both inoculated and control plants after BPH infestation. The relA-SpoT like protein RSH4 (LOC_Os05g06920.1) was down-regulated in both treated and control plants after BPH infestation; however, it was upregulated in BPH-infested plants exposed to bacteria compared with BPH-infested plants unexposed to bacteria (Figure 3).

Following bacterial treatment and BPH infestation, JA, ET and auxin-related genes were differentially expressed in the signaling pathway (Figure 3). Three genes controlling JA synthesis including lipoxygenase 2.2 (LOC_Os12g37320.1), lipoxygenase protein (LOC_Os12g37350.1) and lipoxygenase 2.1 (LOC_Os12g37260.1) were remarkably upregulated by bacterial treatment in plants infested with BPH.

### Oxidative Stress Response to *B. velezensis* YC7010 and BPH Infestation in Rice

Three oxidative stress-related genes including a peroxiredoxin (LOC_Os06g09610.1), a catalase (LOC_Os02g02400.1) and a peroxidase (LOC_Os07g48040.1) were upregulated in response to bacterial treatment of BPH-infested plants compared with BPH-infested plants without bacterial inoculation (Figure 3). However, another peroxidase gene (LOC_Os02g14440.1) was downregulated. Even with the inoculation of only bacteria, all these genes were upregulated without infestation of BPH.

### Photosynthesis and Carbohydrate Metabolism Changes by *B. velezensis* YC7010 and BPH Infestation in Rice

**FIGURE 4:**
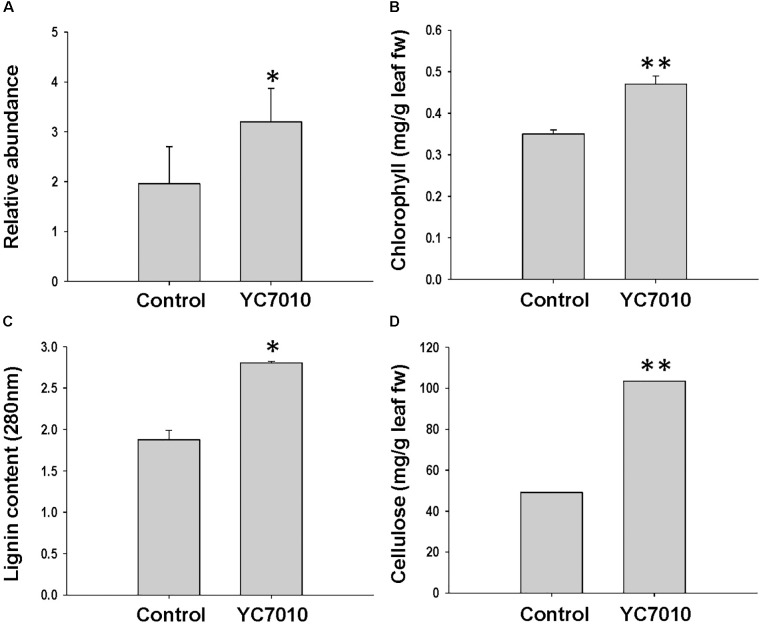
Measurement of **(A)** chlorophyll derivative; **(B)** chlorophyll; **(C)** lignin; and **(D)** cellulose content in rice plants treated with *B. velezensis* YC7010. Bacterial suspension (2 × 10^7^ CFU/mL) was drenched to the rhizosphere of 3-week-old rice plants. A total of 10 s instar nymphae per plant seedling were used at 5 days after treatment. Chlorophyll derivatives were measured at 48 hpi. Chlorophyll, lignin and cellulose contents were measured at 5 days after infestation. The samples were collected from 15 bacteria treated and untreated plants. All experiments were conducted three times. Data were analyzed by independent Student’s *t*-tests. Bars represent mean values ± standard error (SE). Statistical significance for treatment effects is marked ^∗^*P* < 0.05, ^∗∗^*P* < 0.01.

The functional category of photosynthesis presents the highest number of DEGs (Supplementary Figure S2). Most of the DEGs assigned to photosynthesis were upregulated by bacterial treatment regardless of BPH infestation and were downregulated by BPH infestation (Figure 3), which was confirmed by metabolomics analysis. The relative abundance of chlorophyll derivatives and chlorophyll content was higher in treated plants than in untreated plants after BPH infestation (Supplementary Figure S3 and Figures 3, 4A,B). The carbohydrate (CHO) metabolism was also affected by the inoculation of YC7010 and BPH infestation. DEGs related to CHO metabolism such as a sucrose synthase (LOC_Os06g09450.2), a beta-amylase (LOC_Os03g04770.1), and three uncharacterized glycosyltransferases (LOC_Os03g59430.2, LOC_Os07g10840.1 and LOC_Os08g38710.1) and a trehalose-6-phosphate synthase (LOC_Os02g54820.1) were downregulated following BPH-infestation of treated plants compared with plants without bacterial inoculation (Figure 3).

### Metabolites and Cell Wall Modification Induced by *B. velezensis* YC7010 and BPH Infestation

**FIGURE 5:**
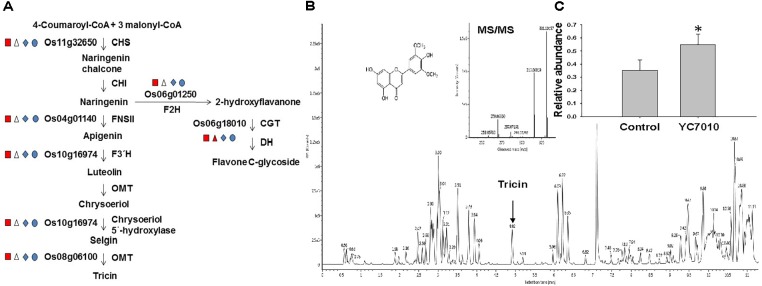
Flavonoid pathways induced in the rice plant by *B. velezensis* YC7010. **(A)** Biosynthetic pathway of tricin and flavone *C*-glycoside. Enzyme abbreviations used are as follows: CHS, chalcone synthase; CHI, chalcone isomerase; F3′H, flavonoid 3′-hydroxylase; FNSII, flavone synthase II; OMT, *O*-methyltransferase; F2H, flavanone 2-hydroxylase; CGT, *C*-glucosyltransferase; and DH, dehydratase. Square, triangle, diamond and oval represent representative genes were compared between Control_Control + BPH, YC7010_YC7010 + BPH, Control_YC7010 and Control + BPH_YC7010 + BPH, respectively. Red = log2FC ≤ –1 Blue = log2FC ≥ 1 and white = log2FC ≤ 1 or log2FC ≥ –1. **(B)** Tricin content was analyzed using Waters Acquity UPLC-Q-TOF. Data were analyzed by independent Student’s *t*-tests. Bars represent mean values ± standard error (SE). Statistical significance for treatment effects is marked ^∗^*P* < 0.05. **(C)** Measurement of tricin content in rice plants treated with YC7010. Bacterial suspension (2 × 10^7^ CFU/mL) was drenched to the rhizosphere of 3-week-old rice plants. A total of 10 s instar nymphae per plant seedling were placed at 5 days after treatment. Tricin contents were measured at 48 h after infestation. The samples were collected from 15 bacteria treated and untreated plants. All experiments were conducted three times.

**FIGURE 6:**
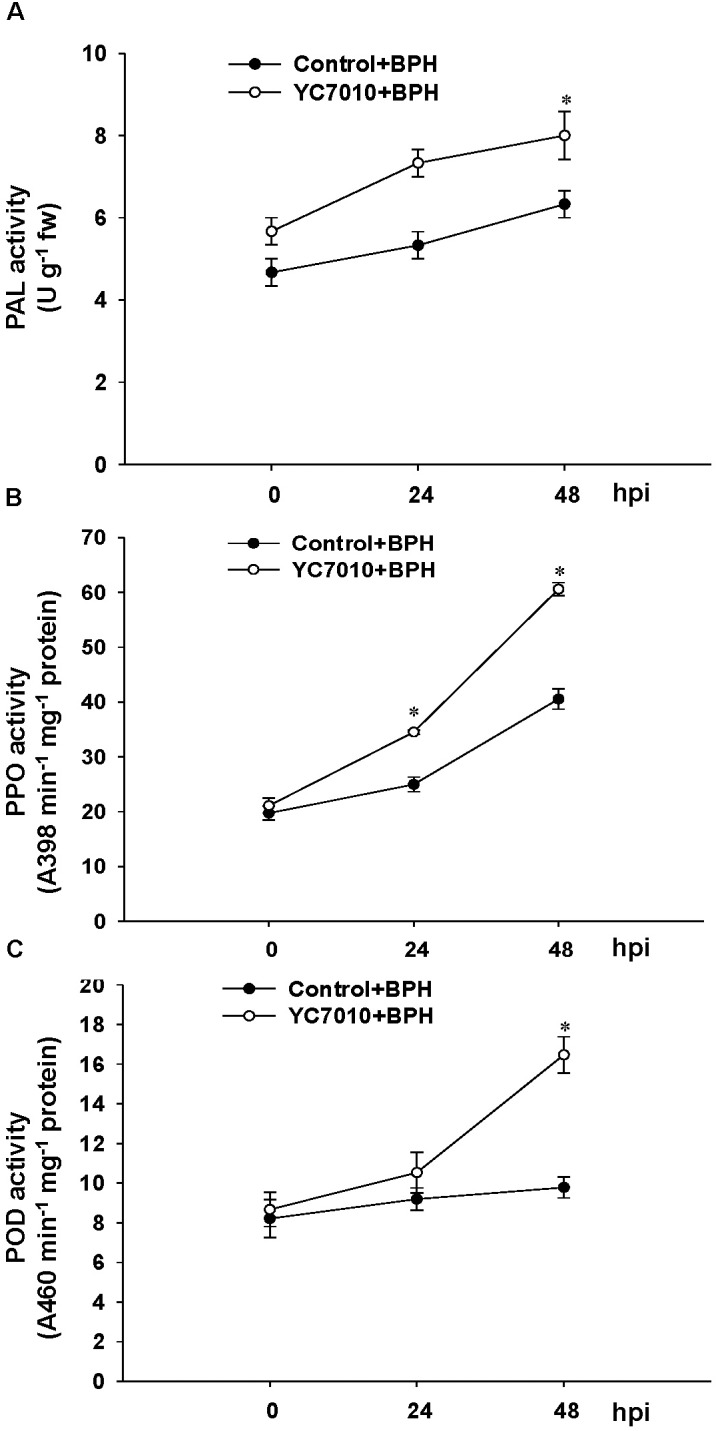
Induction of plant defense enzyme activities by *B. velezensis* YC7010 in rice plant. **(A)** Phenylalanine ammonia-lyase (PAL), **(B)** polyphenol oxidase (PPO), and **(C)** Peroxidase (POD). Bacterial suspension (2 × 10^7^ CFU/mL) was drenched to the rhizosphere of 3-weeks old rice plants while 10 mM MgSO_4_ solution was used as a negative control. Five days later, plants were infested with 10 s instar nymphae per plant seedling and covered using fine, light-transmitting mesh. For enzyme assay, the leaves of 15 treated and untreated rice plants were collected as a mixed samples at 0, 24, and 48 hpi of BPH and the experiment was conducted in triplicate with similar results. Data were analyzed by independent Student’s *t*-tests. Statistical significance for treatment effects is marked ^∗^*P* < 0.05. Bars represent mean values ± standard error (SE).

The colonization of rice roots by YC7010 resulted in enhanced expression of terpene and chalcone synthase genes (LOC_Os08g04500.1 and LOC_Os11g32650.1). Transcriptome analysis showed that flavonoids such as tricin and *C*-glycosyl flavone biosynthesis genes were upregulated following BPH infestation in bacteria-treated rice (Figure 5A). Metabolome analysis further confirmed a higher tricin content in infected plants compared with uninfected control plants (Figures 5B,C). This study also showed that the activities of PAL, PPO, and POD were significantly higher in bacteria-treated rice than in untreated control plants at 48 hpi of BPH (Figures 6A–C). The accumulation of lignin was significantly higher in bacteria treated plants than untreated plants after BPH infestation (Figure 4C). Root drenching with YC7010 enhanced the expression of a cellulose synthesis gene (LOC_Os07g24190.1), which increased the cellulose content (Figures 3, 4D).

### qRT-PCR Analysis of Differentially Expressed Genes

To validate the changes observed in the RNA sequencing data, qRT-PCR analysis of 16 representative genes showed differential expression following the interaction between plants, microbes and insects (Supplementary Figure S4). The pairwise relative expression differences comparing bacteria-treated and untreated control plants were considered for the elucidation of the defense responses induced at 48 hpi of BPH. The qRT-PCR data of these genes were consistent with the RNA sequencing results. Especially, both qRT-PCR and RNA sequencing analyses showed that genes related to jasmonate metabolism (LOC_Os12g37320.1, LOC_Os12g37350.1 and LOC_Os12g37260.1), secondary metabolism (LOC_Os01g63190.1, LOC_Os08g04500.1, LOC_Os04g43800.1, LOC_Os02g41650.3, LOC_Os05g35290.1, LOC_Os02g41630.2, LOC_Os04g43760.1 and LOC_Os11g32650.1), stress (LOC_Os12g14440.1) and photosynthesis (LOC_Os09g36450.1) were up-regulated in plants infected with bacteria compared with control plants after BPH infestation. Similarly, the expression patterns of two oxidation-related genes (LOC_Os02g02400.1 and LOC_Os07g48040.1), and genes involved in cell-wall modification (LOC_Os07g24190.1) in the infected and control plants after BPH infestation were found similar in RNA sequencing and qRT-PCR analyses.

### Isolation and Identification of Compounds With Elicitation of ISR to BPH

**FIGURE 7:**
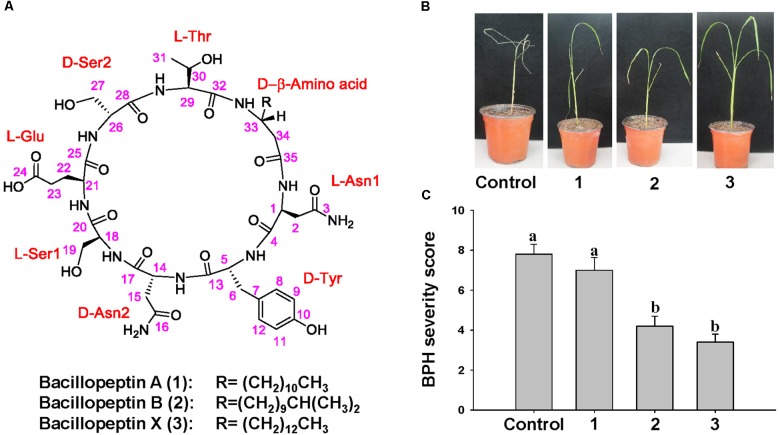
Chemical structure of compounds with ISR activity to BPH isolated from culture broth of *B. velezensis* YC7010. **(A)** Structures of the isolates from *B. velezensis* YC7010. Sterilized seeds were sown on the microwell plates (2 cm × 2 cm/whole) cultivated at growth chamber at 30°C (light; 200 μmol m^-2^ sec^-1^ and 16 h-light/8 h-dark regime). Two-weeks old rice seedlings were treated with 100 μl of bacillopeptin A (1), bacillopeptin B (2) and bacillopeptin X (3) and water was drenched as control. Seedlings were transplanted to plastic pots containing 150 g soil 5 days after treatment. Plants were infested with 15 s instar nymphae per plant seedling and covered using fine and light-transmitting mesh at 3 days after transplant. **(B)** Rice seedlings imaged after BPH infestation. **(C)** BPH severity score was determined at 7 days after infestation. Data were analyzed by one-way ANOVA. Bars represent mean values ± standard error (SE). Means with different letters were significantly different (*P* < 0.05). Each treatment had fives replicates. Experiments were conducted three times.

To determine the chemical structure of compounds to elicit ISR to BPH by YC7010, active compounds were isolated and purified from the culture broth of YC7010. The methanol extract of bacterial culture broth was subjected to multiple chromatographic purification steps including reversed-phase C18 flash column chromatography, silica MPLC, and preparative C18 HPLC to produce three lipopeptides: one new (3) and two known bacillopeptins (1–2) (Figure 7A).

Compounds 1–3 were obtained as pale brown solids. The molecular formulae of 1–3 were determined to be C_46_H_72_N_10_O_16_, C_47_H_74_N_10_O_16_, and C_48_H_76_N_10_O_16_, respectively, based on their HR-TOF-MS (m/z 1043.5016 [M + Na]^+^, 1057.5172 [M + Na]^+^, and 1071.5360 [M + Na]^+^ respectively, positive ESI mode) measurements and confirmed by ^13^C NMR data (Supplementary Figures S5–S13). Compounds 1 and 2 were identified by comparison of their spectroscopic data and physical properties with those reported in the literature, as bacillopeptin A and bacillopeptin B (Kajimura et al., 1995), respectively.

The molecular formula C_48_H_76_N_10_O_16_ of compound 3, deduced from its HR-TOF-MS, was 28 mass units higher than that of the compound 1, and 14 mass units higher than that of the compound 2. Detailed NMR spectroscopic analyses indicated that compounds 1–3 contained identical amino acid sequence and skeleton, but structurally different only in the β-amino acid side chain. The length and type of β-amino acid chains were determined by a combination of the molecular weight and chemical shift of the terminal methyl group. The ^1^H and ^13^C NMR spectra of 1–3 showed the presence of the –(CH_2_)_10_CH_3_, –(CH_2_)_9_CH(CH_3_)_2_, and –(CH_2_)_12_CH_3_ groups, respectively. Thus, the structure of 3 was identified as a novel compound with a β-amino acid chain length of 16 carbons.

To 7 days after infestation with 15 s instar nymphae per plant seedling, control and bacillopeptin A treated plants showed symptoms of stem chlorosis, leaf wilting and even death of the whole plant, whereas plants treated with bacillopeptin B and bacillopeptin X were healthy (Figure 7B). Bacillopeptin X-treated plants were healthier with a severity score of 3.4 compared with bacillopeptin B-treated plants with 4.2. However, bacillopeptin B and bacillopeptin X-treated plants exhibited a significant reduction in BPH infestation compared with bacillopeptin A and untreated control (Figure 7C). The results indicated that bacillopeptin B and bacillopeptin X might induce systemic resistance to BPH.

## Discussion

Systemic resistance induced in plant by certain beneficial PGPR or endophytic bacteria commonly found inside roots against insect pests (Pineda et al., 2010; Van de Mortel et al., 2012; de Oliveira Araujo, 2015). These bacteria with ability to enhance plant growth and development have the potential to be utilized for biological control of numerous insect pests. We have previously identified an endophytic *B. velezensis* YC7010 with anti-microbial, plant growth-promoting, and systemic resistance-inducing activity against insect (Chung et al., 2015; Hossain et al., 2016; Rashid et al., 2017). In this study, our results demonstrated that root drenching of rice with *B. velezensis* YC7010 suspension resulted in the establishment of an ISR against BPH (Figure 1). Valenzuela-Soto et al. (2010) reported that inoculation of tomato roots with growth-promoting *B. subtilis* retarded the development of phloem-feeding insect whitefly by inducing resistance, which is in agreement with our results. However, few reports have been published to date on tritrophic interactions among bacteria, insects and host plants, which were inadequate to elucidate the mechanism of ISR against insects by endophytic bacteria.

RNA sequencing of the bacterial treated and the untreated rice plants provided transcriptome data on the mechanism of resistance conferred by *B. velezensis* YC7010. In our study, several rice genes have been associated with the BPH and bacterial interaction for the first time, which provides information on signal transduction pathways and defense responses elicited by the BPH in rice. We analyzed plants at 48hpi, before the development of visible symptoms, and identified the transcript profiles change in different rice samples (Figure 2 and Supplementary Table S4). In our study, LRR family and Ca^2+^ signaling genes were upregulated in both bacterial treated and control infested plants compared with uninfested plants (Figure 3). The LRR family protein kinases have been implicated to trigger plant defense responses to herbivore attack (Howe and Jander, 2008). The receptor kinases activate downstream signals by inducing the production of reactive oxygen species (ROS) (Wrzaczek et al., 2010). RelA/SpoT stress proteins regulate the expression of chloroplast gene in response to plant defense signals (Givens et al., 2004). In this study, Ca^2+^ signaling, relA-SpoT like protein RSH4 was up-regulated with the BPH infestation in bacteria treated plants compared to control plants, which suggests that the rice defense response to BPH involved Ca^2+^ influx.

The initial step in JA biosynthesis generating 12-oxo-phytodienoic acid (OPDA) from α-linolenic acid (α-LeA) is mediated by lipoxygenase (*LOX*) genes and is involved in the induction of defense responses against wounding and in regulation of the defense gene expression (Stintzi et al., 2001; Wasternack, 2007). Our study identified three lipoxygenase genes, which were upregulated with the BPH infestation in bacteria treated plants compared to control plants (Figure 3). We suggest that bacterial treatment with YC7010 might enhance effective defense response against BPH in rice plants wounded by BPH infestation that might induce biosynthesis of OPDA. Oxidative stress is a general plant reaction to tissue penetration and injury by phloem-sap sucking insects (Kehr, 2006). Proteins and metabolites with redox and anti-oxidative properties are, therefore, essential not only for the maintenance of physiological function during their long lifetime but also for the management of phloem-sap sucking insect infestations. ROS play several crucial physiological roles within plants at low concentrations. However, their effects become detrimental at higher concentrations (Walz et al., 2002; Kehr, 2006). This study showed BPH feeding induces a strong accumulation of ROS scavengers and enzymatic antioxidant systems in bacterial treated than in control rice plant. The accumulation of ROS scavengers allows resistant plants to avoid detrimental effects, suggesting that the bacterial treatment with BPH infestation alters the redox status contributing to resistance mechanisms in rice.

Following BPH infestation of rice leaves, photosynthetic genes were downregulated (Yuan et al., 2005). Several photosynthesis genes were downregulated in both bacteria inoculated and control plants following BPH infestation but those were up-regulated in BPH infested bacteria treated plants compared to BPH infested control plants and chlorophyll derivative as well as chlorophyll content was also higher in bacterial treated than in control plants after BPH infestation, suggesting that the rice photosynthetic apparatus was less damaged during BPH infestation in bacterial treated than in control plant (Figures 3, 4A,B). It was also reported that colonization of tomato roots by growth-promoting rhizobacteria induces resistance against the phloem-feeding insect whitefly via increased expression of photosynthetic genes, consistent with our results (Valenzuela-Soto et al., 2010). The expression of sucrose synthase and beta-amylase expression were downregulated with the BPH infestation in bacteria treated compared to control plants (Figure 3). It was shown that ingestion of a large amount of phloem sap by continuous feeding of BPH resulted in a rapid decline in sucrose content triggering the expression of plant genes such as α-amylase, which catalyzes the conversion of starch to sucrose. This process maintains normal growth of BPH in susceptible rice until the storage starch is exhausted (Yuan et al., 2005; Hao et al., 2008). Continuous feeding by BPH induces inhibition of photosynthesis and reduces the production and translocation of assimilates (Yuan et al., 2005; Wang Y. et al., 2008). Our results suggested that bacterial treatment with YC7010 ensures minimal disruption of the translocation system resulting in reduced loss of sucrose compared with untreated controls. Thus, rice is more resistant to, or tolerant of, the ingestion process by BPH.

The ISR produced during the bacterial and BPH interaction could have benefited from the up regulated expression of a jacalin-related lectin gene which was used as a control agent against both biting-chewing and piercing-sucking pest insects (Al Atalah et al., 2014) (Figure 3). Jasmonate-regulated proteins (JRPs) such as lipoxygenase and a jacalin-related lectin are associated with numerous biological activities and biochemical processes. These include plant secondary metabolism, formation of cell wall structure, stress adaptation and resistance to pathogens and insects (Pauwels et al., 2009; Tong et al., 2012). The colonization of rice roots by YC7010 resulted in enhanced expression of terpene and chalcone synthase genes (LOC_Os08g04500.1 and LOC_Os11g32650.1) which is known to be associated with the defense against BPH (Kamolsukyunyong et al., 2013). The expression of PAL genes and phenol synthesis genes, which act as antifeedants against insect herbivores such as *Operophtera brumata* (L.) in *Salix* leaves was enhanced by bacterial treatment (Simmonds, 2003) (Figure 3). RNA sequencing analysis showed that flavonoids such tricin and *C*-glycosyl flavone biosynthesis genes were upregulated with the BPH infestation in bacteria treated compared to control plants (Figure 5). The high C-glycosyl flavones-producing genes in sweet corn protected ears against fall armyworm and *Euxesta stigmatias* damage (Nuessly et al., 2007). Tricin was shown to confer resistance against BPH in rice and has been recently reported as the initial step in lignification of monocots (Bing et al., 2007; Lan et al., 2015). In addition, fortification of the cell-wall in bacteria-treated plants was further promoted by the enhanced expression of the SA synthesis-related gene PAL. PAL is involved in the biosynthesis of lignins and plays a considerable role in resistance to small brown planthopper (SBPH) (Pellegrini et al., 1994; Polle et al., 1994; Duan et al., 2014). However, our study also showed the activity of PAL, PPO, and POD were higher in bacterial treated plants than in control plants after BPH infestation (Figure 6). POD and PPO are also known to be involved in the synthesis of lignin and other oxidative phenols that contribute to the formation of defense barriers for reinforced cell structure (Avdiushko et al., 1993) consistent with the increased level of accumulated lignin in bacterial-treated plants (Figure 4C). It has been reported that the changes of peroxidase biosynthesis and cellulose synthase strengthened the physical barriers of rice, which increased resistance against SBPH and rice strive virus (RSV) (Zheng et al., 2013). The upregulation of beta-glucosidase gene (LOC_Os03g53800.3) in bacterial treated plant activates defense responses against both sucking and chewing insects (Wang X. et al., 2005) (Figure 3). Fatty-acid chain lengths of n-C14, iso-C15, iso-C16, and n-C15 of bacillopeptins A (compound 1), B (compound 2), C, and B_1_, respectively, were previously reported from *B. subtilis* FR-2 (Kajimura et al., 1995) and *Bacillus amyloliquefaciens* SH-B74 (Ma et al., 2014). However, the compound 3 with fatty-acid chain length of n-C16 was isolated from a new strain of *B. velezensis* YC7010 (Figure 7). The nature of the β-amino fatty acid residue indicated that compound 3 was a novel metabolite belonging to the bacillopeptin family and designated as bacillopeptin X. Another endophytic bacterium *B. amyloliquefaciens* also elicited ISR in plants against fall armyworms by producing lipopeptides (Li et al., 2015). Many *Bacillus* species are well known to produce a variety of lipopeptides such as iturin, surfactin, and fengycin. Genomic analysis and biosynthesis of these peptides have been reported (Ongena and Jacques, 2008). However, no genetic study of these bacillopeptins has been reported yet. It is necessary to further elucidate the underlying mechanisms to develop them as potential tools for biocontrol of insect pests.

In summary, the root treatment with *B. velezensis* YC7010 activated ISR against BPH in rice by producing cyclic lipopeptides including a new bacillopeptin X. The results indicate that the key defense mechanism in bacteria-treated rice plants was dependent on SA and JA pathways. Especially, the chemical change in flavonoids, tricin and physical strengthening of cell walls via increased cellulose and lignin contents may play a critical role comprehensively in ISR to BPH. Further analysis showed that hypersensitive reaction (HR) may not be the mechanism underlying the resistance to BPH in plants infected with bacteria, which resulted in an increase in peroxidase expression and downregulation of LRR receptor-like protein kinase. To the best of our knowledge, this is the first report elucidating the mechanism of ISR against BPH by an endophytic PGPR. In this aspect, the results of this study facilitate the development of biological control strategies against insect pests using beneficial endophytic bacteria.

## Author Contributions

YC, MR, H-JK, S-IY, S-SM, and YK planned and designed the study. MR, H-AY, H-JK, and MM performed the study. MR, MM, and YK analyzed the data. YC, MR, S-IY, S-SM, and YK wrote the manuscript.

## Supplementary Material

The Supplementary Material for this article can be found online at: https://www.frontiersin.org/articles/10.3389/fpls.2018.01904/full#supplementary-material

## Conflict of Interest Statement

The authors declare that the research was conducted in the absence of any commercial or financial relationships that could be construed as a potential conflict of interest.

## Footnotes

1 http://phytozome.jgi.doe.gov/pz/portal.html#!info?alias=Org_Osativa

2 http://mapman.gabipd.org/web/guest/mapman

3 http://www.bio-rad.com/
