# Supplementary material for: Bacillus velezensis YC7010 Enhances Plant Defenses Against Brown Planthopper Through Transcriptomic and Metabolic Changes in Rice
Source: Front Plant Sci. 2018 Dec 21;9:1904. doi: 10.3389/fpls.2018.01904 (PMC6308211; doi:10.3389/fpls.2018.01904)
Supplement: Supplementary file 14 [file Table_1.DOCX]

**Table S1.** Properties of transcriptome of bacterial treated and untreated rice seedlings were infested with (BPH) or without in three biological replicates

| Treatments | Number of reads | | | Read length (bp) |
| --- | --- | --- | --- | --- |
|  | 1st | 2nd | 3rd |  |
| Control | 6475798 | 6270328 | 6249993 | 101 |
| Control + BPH | 6613270 | 6885765 | 6734502 | 101 |
| YC7010 | 6518313 | 6275791 | 6152300 | 101 |
| YC7010 + BPH | 6269475 | 5989559 | 7202910 | 101 |
