# Supplementary material for: Bacillus velezensis YC7010 Enhances Plant Defenses Against Brown Planthopper Through Transcriptomic and Metabolic Changes in Rice
Source: Front Plant Sci. 2018 Dec 21;9:1904. doi: 10.3389/fpls.2018.01904 (PMC6308211; doi:10.3389/fpls.2018.01904)
Supplement: Supplementary file 15 [file Table_2.DOCX]

**Table S2**. Primers used in this study for quantitative RT-PCR

| Primer names | Forward primer (5' - 3') | Reverse primer (5' - 3') |
| --- | --- | --- |
| LOC_Os01g63190.1 | ATGGTTATACCTTGGTGTTCGTC | TCATTCACTTCTATCATTGGGC |
| LOC_Os08g04500.1 | ATGGCAACCTCTGTTCCG | GAAGGTAAGAAAGTAATCGCCC |
| LOC_Os04g43800.1 | ATGGAGTGCGAGAACGGG | CTCCTCCACCATGCGCTT |
| LOC_Os02g41650.3 | ATGAACAGCATGATGAACGG | TTGATGCGGACGAGCATC |
| LOC_Os05g35290.1 | ATGGAGTGCGAGACTGGC | GTACTCCGCCACCATCCTC |
| LOC_Os02g41630.2 | ATGGACAACGCCCGTCTC | GGGTTGGCGAGGTACTGG |
| LOC_Os04g43760.1 | ATGGCAAGAAGGTGGACG | CATCACCTCGCAGAACACG |
| LOC_Os11g32650.1 | ATGGTCGAGCTCAAGGAGAA | TCCGTCAGGTGCATGTACC |
| LOC_Os12g37320.1 | ATGCAGGTACAGGGCATAACC | CTCCGTGTGCCTCTCTGTACT |
| LOC_Os12g37350.1 | ATGGCAAGCTGAAGCTAACG | ATTCGTTTGCACCTCGTTC |
| LOC_Os12g37260.1 | ATGGATGGCAAAGGCTCC | GATCCAAGAGTTGCAGTGGAA |
| LOC_Os12g14440.1 | ATGGCTGATCCCAGCAAG | CTGCCGATTCCGTCATATACT |
| LOC_Os09g36450.1 | ATGCTGCTGTTCGTGATTG | TCTTGGAAGTCACCGAGTTG |
| LOC_Os07g24190.1 | ATGGGCAGATGGTTGATGAC | TTCCAGCTCTCCATCCTCTC |
| LOC_Os02g02400.1 | ATGGATCCTTGCAAGTTCCG | CTTCTCGATCAGGTGGTAGTCC |
| LOC_Os07g48040.1 | ATGGCTTCTGCCTCTTCTCTT | AAGCAGTCGTGGAAGTGCA |
| OsActin | ACCCCATCGAGCATGGTATCGTCA | CAGCCTTGGCAATCCACATCTGCT |
