# Supplementary material for: Bacillus velezensis YC7010 Enhances Plant Defenses Against Brown Planthopper Through Transcriptomic and Metabolic Changes in Rice
Source: Front Plant Sci. 2018 Dec 21;9:1904. doi: 10.3389/fpls.2018.01904 (PMC6308211; doi:10.3389/fpls.2018.01904)
Supplement: Supplementary file 16 [file Table_3.DOCX]

**Table S3.** Methods for the extraction and isolation of bacterial compounds

| **Works** | **Details procedure** |
| --- | --- |
| Extraction and isolation | Incubated culture media (20 L) of *B. velezensis* YC7010 was concentrated to 3L and extracted with methanol (1000 mL) yielding a brown syrup (241 g). The concentrate was chromatographed on a C18 flash column using a 10% stepwise gradient elution of increasing MeOH concentration in H_2_O (2.0 L each) to afford eleven fractions (A to K: 172.8 g, 23.4 g, 13.4 g, 8.3 g, 2.9 g, 1.5 g, 1.0 g, 0.9 g, 0.8 g, 0.7 g, and 2.4 g, respectively). The ISR active fraction I (800 mg) was fractionated by silica MPLC eluting with EtOAC-MeOH (1:0, 1:1, and 0:1, 500 mL each) to yield three subfractions (I1-I3). Subfraction I2 (380 mg, 1:1 EtOAC/MeOH eluate) was purified by repeated preparative C18 HPLC to yield compounds **1** (10.0 mg, *t*_R_ 45.0 min), **2** (13.3 mg, *t*_R_ 49.0 min), and **3** (10.0 mg, *t*_R_ 56.5 min).  **Bacillopeptin A (1):** A brown solid; ^1^H NMR (DMSO-*d_6_*, 400 MHz) δ12.07 (1H, br s, L-Glu, 24-OH), 9.16 (1H, s, D-Tyr, 10-OH), 8.27 (1H, d, *J* = 7.2 Hz, D-Tyr, 5-NH), 8.12 (1H, d, *J* = 7.6 Hz, D-Ser2, 26-NH), 7.99 (1H, m, D-Asn2, 14-NH), 7.96 (1H, m, L-Glu, 21-NH), 7.88 (1H, d, *J* = 6.0 Hz, L-Asn1, 1-NH), 7.67 (1H, d, *J* = 6.8 Hz, L-Thr, 29-NH), 7.55 (1H, d, *J* = 7.6 Hz, L-Ser1, 18-NH), 7.29 (2H, br s, β-amino acid, 33-NH and D-Asn2, 16-NH_2_), 7.25 (1H, br s, L-Asn1, 3-NH_2_), 7.0 (2H, d, *J* = 8.0 Hz, D-Tyr, H-8 and H-12), 6.93 (2H, br s, L-Asn1, 3-NH_2_ and D-Asn2, 16-NH_2_), 6.62 (2H, d, *J* = 8.0 Hz, D-Tyr, H-9 and H-11), 4.91 (1H, m, D-Ser2, 27-OH), 4.86 (1H, m, L-Ser1, 19-OH), 4.80 (1H, br s, L-Thr, 30-OH), 4.46 (1H, m, D-Asn2, H-14), 4.38 (1H, m, L-Asn1, H-1), 4.33 (1H, m, D-Ser2, H-26), 4.25 (1H, m, L-Ser1, H-18), 4.23 (1H, m, D-Tyr, H-5), 4.19 (1H, m, L-Glu, H-21), 4.06 (1H, m, L-Thr, H-30), 4.04 (1H, m, L-Thr, H-29), 3.96 (1H, m, β-amino acid, H-33), 3.59 (4H, m, L-ser1, H-19 and D-Ser2, H-27), 2.94 (1H, dd, *J* = 14.0, 4.5 Hz, D-Tyr, H_a_-6), 2.68 (1H, dd, *J* = 14.0, 9.9 Hz, D-Tyr, H_b_-6), 2.57 (1H, dd, *J* = 15.3, 5.4 Hz, D-Asn2, H_a_-15), 2.50 (1H, overlapped with solvent, D-Asn2, H_b_-15), 2.33 (1H, m, L-Asn1, H_a_-2), 2.29 (2H, m, β-amino acid, H-34), 2.28 (2H, m, L-Glu, H-23), 2.20 (1H, m, L-Asn1, H_b_-2), 1.92 (1H, m, L-Glu, H_a_-22), 1.82 (1H, m, L-Glu, H_b_-22), 1.40 (1H, m, β-amino acid, H_a_-36), 1.35 (1H, m, β-amino acid, H_b_-36), 1.26-1.15 (18 H, m, β-amino acid, H-37 to H-45), 1.0 (3H, d, *J* = 6.0 Hz, L-Thr, H-31), 0.83 (3H, t, *J* = 7.2 Hz, β-amino acid, H-46) ppm; ^13^C NMR (100 MHz, DMSO-*d_6_*) δ 174.1 (C-24), 172.3 (C-4), 171.7 (C-25), 171.41 (C-16), 171.35 (C-3), 171.27 (C-13), 171.2 (C-17), 171.0 (C-35), 170.5 (C-28), 170.2 (C-20), 169.3 (C-32), 155.7 (C-10), 129.9 (C-8 and C-12), 128.0 (C-7), 115.0 (C-9 and C-11), 65.8 (C-30), 61.3 (C-19), 61.0 (C-27), 58.6 (C-29), 55.6 (C-5), 55.2 (C-26), 55.0 (C-18), 52.8 (C-21), 50.7 (C-14), 50.2 (C-1), 46.1 (C-33), 40.6 (C-34), 36.7 (C-15), 36.5 (C-2), 35.5 (C-6), 33.8 (C-36), 31.3 (C-44), 30.1 (C-23), 29.1 (C-43), 29.07 (C-41and C-42), 29.06 (C-40), 28.7 (C-39), 28.68 (C-38), 26.8 (C-22), 25.2 (C-37), 22.1 (C-45), 20.0 (C-31), 14.0 (C-46); HRTOFMS (positive ESI mode) *m/z* 1043.5016 [M + Na]^+^ (calcd for C_46_H_72_N_10_O_16_+Na, 1043.5025).  **Bacillopeptin B (2):** A brown solid; ^1^H NMR (DMSO-*d_6_*, 400 MHz) δ 12.06 (1H, br s, L-Glu, 24-OH), 9.16 (1H, s, D-Tyr, 10-OH), 8.27 (1H, d, *J* = 7.2 Hz, D-Tyr, 5-NH), 8.12 (1H, d, *J* = 7.6 Hz, D-Ser2, 26-NH), 7.99 (1H, d, *J* = 6.8 Hz, D-Asn2, 14-NH), 7.96 (1H, d, *J* = 6.0 Hz, L-Glu, 21-NH), 7.88 (1H, d, *J* = 6.0 Hz, L-Asn1, 1-NH), 7.67 (1H, d, *J* = 6.8 Hz, L-Thr, 29-NH), 7.54 (1H, d, *J* = 7.6 Hz, L-Ser1, 18-NH), 7.30 (1H, br s, D-Asn2, 16-NH_2_), 7.28 (1H, m, β-amino acid, 33-NH), 7.25 (1H, br s, L-Asn1, 3-NH_2_), 6.99 (2H, d, *J* = 8.4 Hz, D-Tyr, H-8 and H-12), 6.94 (2H, br s, L-Asn1, 3- NH_2_ and D-Asn2, 16-NH_2_), 6.62 (2H, d, *J* = 8.4 Hz, D-Tyr, H-9 and H-11), 4.90 (1H, m, D-Ser2, 27-OH), 4.85 (1H, m, L-Ser1, 19-OH), 4.80 (1H, m, L-Thr, 31-OH), 4.46 (1H, m, D-Asn2, H-14), 4.38 (1H, m, L-Asn1, H-1), 4.34 (1H, m, D-Ser2, H-26), 4.23 (1H, m, L-Ser1, H-18), 4.20 (1H, m, D-Tyr, H-5), 4.18 (1H, m, L-Glu, H-21), 4.05 (1H, m, L-Thr, H-29), 4.04 (1H, m, L-Thr, H-30), 3.97 (1H, m, β-amino acid, H-33), 3.58 (2H, m, D-Ser2, H-27), 3.57 (2H, m, L-Ser1, H-19), 2.94 (1H, dd, *J* = 13.2, 5.6 Hz, D-Tyr, H_a_-6), 2.68 (1H, dd, *J* = 13.2, 9.9 Hz, D-Tyr, H_b_-6), 2.55 (1H, dd, *J* = 15.3, 5.4 Hz, D-Asn2, H_a_-15), 2.50 (1H, overlapped with solvent, D-Asn2, H_b_-15), 2.28 (2H, m, L-Glu, H_a_-23 and L-Asn1, H_a_-2), 2.26 (1H, m, β-amino acid, H_a_-34), 2.21 (1H, m, L-Glu H_b_-23), 2.19 (1H, m, β-amino acid, H_b_-34), 2.18 (1H, m, L-Asn1, H_b_-2), 1.91 (1H, m, L-Glu, H_a_-22), 1.81 (1H, m, L-Glu, H_b_-22), 1.46 (1H, m, β-amino acid, H-45), 1.40 (1H, m, β-amino acid, H_a_-36), 1.36 (1H, m, β-amino acid, H_b_-36), 1.26-1.11 (14 H, m, β-amino acid, H-37 to H-43 and H_a_-44), 1.11 (1H, m, β-amino acid, H_b_-44), 1.0 (3H, d, *J* = 6.0 Hz, L-Thr, H-31), 0.82 (6H, d, *J* = 6.4 Hz, β-amino acid, H-46 and H-47) ppm; ^13^C NMR (100 MHz, DMSO-*d_6_*) δ 174.0 (C-24), 172.3 (C-4), 171.7 (C-25), 171.41 (C-16), 171.35 (C-3), 171.3 (C-13), 171.2 (C-17), 171.0 (C-35), 170.5 (C-28), 170.2 (C-20), 169.4 (C-32), 155.7 (C-10), 129.9 (C-8 and C-12), 128.0 (C-7), 115.0 (C-9 and C-11), 65.8 (C-30), 61.3 (C-19), 61.0 (C-27), 58.6 (C-29), 55.6 (C-5), 55.2 (C-26), 55.0 (C-18), 52.7 (C-21), 50.7 (C-14), 50.2 (C-1), 46.0 (C-33), 41.0 (C-34), 38.5 (C-44), 36.6 (C-15), 36.2 (C-2), 36.0 (C-6), 33.8 (C-36), 30.8 (C-23), 29.3 (C-42), 29.09 (C-40 and C-41), 28.75 (C-39), 28.70 (C-38), 27.4 (C-45), 26.8 (C-22 and C-43), 25.2 (C-37), 22.6 (C-46 and C-47), 20.0 (C-31); HRTOFMS (positive ESI mode) *m/z* 1057.5172 [M + Na]^+^ (calcd for C_47_H_74_N_10_O_16_+Na, 1057.5182).  **Bacillopeptin X (3):** A brown solid; ^1^H NMR (DMSO-*d_6_*, 400 MHz) δ 12.07 (1H, br s, L-Glu, 24-OH), 9.15 (1H, s, D-Tyr, 10-OH), 8.27 (1H, d, *J* = 7.2 Hz, D-Tyr, 5-NH), 8.12 (1H, d, *J* = 7.6 Hz, D-Ser2, 26-NH), 7.99 (1H, m, D-Asn2, 14-NH), 7.96 (1H, m, L-Glu, 21-NH), 7.88 (1H, d, *J* = 6.0 Hz, L-Asn1, 1-NH), 7.67 (1H, d, *J* = 6.8 Hz, L-Thr, 29-NH), 7.55 (1H, d, *J* = 7.6 Hz, L-Ser1, 18-NH), 7.29 (2H, m, β-amino acid, 33-NH and D-Asn2, 16-NH_2_), 7.25 (1H, br s, L-Asn1, 3-NH_2_), 7.0 (2H, d, *J* = 8.0 Hz, D-Tyr, H-8 and H-12), 6.93 (2H, br s, L-Asn1, 3-NH_2_ and D-Asn2, 16-NH_2_), 6.62 (2H, d, *J* = 8.0 Hz, D-Tyr, H-9 and H-11), 4.91 (1H, t, *J* = 5.0 Hz, D-Ser2, 27-OH), 4.85 (1H, t, *J* = 5.0 Hz, L-Ser1, 19-OH), 4.80 (1H, d, *J* = 5.2 Hz, L-Thr, 30-OH), 4.46 (1H, m, D-Asn2, H-14), 4.38 (1H, m, L-Asn1, H-1), 4.33 (1H, m, D-Ser2, H-26), 4.25 (1H, m, L-Ser1, H-18), 4.23 (1H, m, D-Tyr, H-5), 4.19 (1H, m, L-Glu, H-21), 4.06 (1H, m, L-Thr, H-30), 4.04 (1H, m, L-Thr, H-29), 3.96 (1H, m, β-amino acid, H-33), 3.59 (4H, m, L-Ser1, H-19 and D-Ser2, H-27), 2.94 (1H, dd, *J* = 13.2, 4.5 Hz, D-Tyr, H_a_-6), 2.68 (1H, dd, *J* = 13.2, 9.9 Hz, D-Tyr, H_b_-6), 2.57 (1H, dd, *J* = 15.3, 5.4 Hz, D-Asn2, H_a_-15), 2.50 (1H, overlapped with solvent, D-Asn2, H_b_-15), 2.33 (1H, m, L-Asn1, H_a_-2), 2.29 (2H, m, β-amino acid, H-34), 2.28 (2H, m, L-Glu, H-23), 2.20 (1H, m, L-Asn1, H_b_-2), 1.92 (1H, m, L-Glu, H_a_-22), 1.82 (1H, m, L-Glu, H_b_-22), 1.40 (1H, m, β-amino acid, H_a_-36), 1.35 (1H, m, β-amino acid, H_b_-36), 1.26-1.15 (20 H, m, β-amino acid, H-5 to H15), 0.83 (3H, t, *J* = 7.2 Hz, β-amino acid, H-16) ppm; ^13^C NMR (100 MHz, DMSO-*d_6_*) δ 174.0 (C-24), 172.3 (C-4), 171.7 (C-25), 171.41 (C-16), 171.34 (C-3), 171.27 (C-13), 171.2 (C-17), 171.0 (C-35), 170.5 (C-28), 170.2 (C-20), 169.3 (C-32), 155.7 (C-10), 129.9 (C-8 and C-12), 128.0 (C-7), 115.0 (C-9 and C-11), 65.8 (C-30), 61.3 (C-19), 61.0 (C-27), 58.6 (C-29), 55.6 (C-5), 55.2 (C-26), 55.0 (C-18), 52.8 (C-21), 50.7 (C-14), 50.2 (C-1), 46.1 (C-33), 40.6 (C-34), 36.7 (C-15), 36.5 (C-2), 35.5 (C-6), 33.8 (C-36), 31.3 (C-46), 30.1 (C-23), 29.12 (C-43, C-44, and C-45), 29.06 (C-40, C-41, and C-42), 28.7 (C-39), 28.69 (C-38), 26.8 (C-22), 25.2 (C-37), 22.1 (C-47), 20.0 (C-31), 14.0 (C-48); HRTOFMS (positive ESI mode) *m/z* 1071.5360 [M + Na]^+^ (calcd for C_48_H_76_N_10_O_16_+Na, 1071.5338) |
