# Supplementary material for: Bacillus velezensis YC7010 Enhances Plant Defenses Against Brown Planthopper Through Transcriptomic and Metabolic Changes in Rice
Source: Front Plant Sci. 2018 Dec 21;9:1904. doi: 10.3389/fpls.2018.01904 (PMC6308211; doi:10.3389/fpls.2018.01904)
Supplement: Supplementary file 19 [file Table_6.DOCX]

**Table S6.** GO enrichment were analysed in all comparisons. ‘-’ indicates the absence of any enriched GO terms.

| Category | GO ID | GO Terms | Number of DEG | | | |
| --- | --- | --- | --- | --- | --- | --- |
|  |  |  | Control_  Control + BPH | YC7010_YC7010 + BPH | Control_  YC7010 | Control +BPH_  YC7010 + BPH |
| Biological Process | GO:0005975 | Carbohydrate metabolic process | 4 | 1 | - | 1 |
|  | GO:0006091 | Generation of precursor metabolites and energy | 5 | 5 | 6 | - |
|  | GO:0006139 | Nucleobase-containing compound metabolic process | 7 | - | 1 | 4 |
|  | GO:0006629 | Lipid metabolic process | 1 | - | 2 | 1 |
|  | GO:0006810 | Transport | 2 | - | - | 2 |
|  | GO:0007275 | Multicellular organismal development | 1 | - | - | 2 |
|  | GO:0008219 | Cell death | 1 | - | - | 1 |
|  | GO:0009791 | Post-embryonic development | 1 | - | - | - |
|  | GO:0015979 | Photosynthesis | 5 | 5 | 6 | - |
|  | GO:0019538 | Protein metabolic process | 9 | 7 | 3 | - |
|  | GO:0019725 | Cellular homeostasis |  |  | 1 | 2 |
|  | GO:0055085 | Transmembrane transport | 2 | - | - | - |
|  | GO:0055114 | Oxidation-reduction process | - | 5 | - | - |
| Cellular Component | GO:0005737 | Cytoplasm | 37 | 18 | 25 | 30 |
|  | GO:0009579 | Thylakoid |  | 1 |  |  |
| Molecular Function | GO:0000166 | Nucleotide binding | 8 | 4 | 4 | 7 |
|  | GO:0016705 | Oxidoreductase activity, acting on paired donors, with incorporation or reduction of molecular oxygen | - | - | - | 1 |
|  | GO:0016772 | Transferase activity, transferring phosphorus-containing groups | - | - | 4 | 2 |
|  | GO:0016788 | Hydrolase activity, acting on ester bonds | - | - | - | 1 |
